# Subcellular Microanatomy by 3D Deconvolution Brightfield Microscopy: Method and Analysis Using Human Chromatin in the Interphase Nucleus

**DOI:** 10.1155/2012/848707

**Published:** 2012-01-24

**Authors:** Paul Joseph Tadrous

**Affiliations:** Department of Histopathology, Northwick Park Hospital, Watford Road, London HA1 3UJ, UK

## Abstract

Anatomy has advanced using 3-dimensional (3D) studies at macroscopic (e.g., dissection, injection moulding of vessels, radiology) and microscopic (e.g., serial section reconstruction with light and electron microscopy) levels. This paper presents the first results in human cells of a new method of subcellular 3D brightfield microscopy. Unlike traditional 3D deconvolution and confocal techniques, this method is suitable for general application to brightfield microscopy. Unlike brightfield serial sectioning it has subcellular resolution. Results are presented of the 3D structure of chromatin in the interphase nucleus of two human cell types, hepatocyte and plasma cell. I show how the freedom to examine these structures in 3D allows greater morphological discrimination between and within cell types and the 3D structural basis for the classical “clock-face” motif of the plasma cell nucleus is revealed. Potential for further applications discussed.

## 1. Introduction

Anatomical research requires methods for seeing structure in 3D such as the traditional macroscopic methods of dissection or making vascular casts by injection moulding [1, 2]. At the microscopic level, while equivalent techniques at the microscopic level do exist [3], it is more common for 3D microscopy to employ serial section reconstruction at light and electron microscope levels [4, 5] or some form of optical sectioning microscopy such as confocal or deconvolution fluorescence microscopy [6, 7].

In order to overcome some of the limitations of traditional deconvolution and confocal microscopy (which are generally restricted to fluorescent preparations with a few special case exceptions, especially if subcellular detail is required), recently a new method of 3D deconvolution microscopy has been published [8]. This method was designed specifically for brightfield microscopy to make visible routinely stained histological preparations with subcellular 3D resolution using an ordinary light microscope and results on yeast cells were presented [8].

In this paper I present, for the first time, results using this new method applied to human cells, specifically hepatocytes and plasma cells. I show how the method reveals the interphase nuclear chromatin meshwork in 3D. The results are analysed quantitatively with texture analysis and qualitatively to demonstrate the utility of this method in the study of cell and tissue structure.

## 2. Materials and Methods

### 2.1. Tissue Preparation

The cells analysed in this study were imaged from a formalin-fixed paraffin-embedded 10-micron-thick section of human liver tissue which was stained with Williams progressive haematoxylin (only) according to standard diagnostic laboratory protocols. The tissue was acquired from a surgical hepatic segmental resection specimen as surplus to diagnostic requirements with the understanding and consent of the patient and in accordance with a protocol approved by the responsible ethics committee (Mid and South Buckinghamshire REC ref. 07/H0607/92).

### 2.2. Microscopy and Deconvolution

The tissue section was imaged under oil immersion bright-field light microscopy using a ×100 objective lens and a standard diagnostic microscope coupled to a focus-control motor system that allowed images to be acquired at a series of focal planes to produce a 3D “*Z*-stack” of optical images of each cell under study. The image volumes used in this study are all 256 × 256 × 256 voxel cubes and the voxels are isometric with each side of a voxel (*X*, *Y*, and *Z*) equivalent to a length of 81.74 nm. The details of the apparatus were as described in a previous publication [8]. In this way a *Z*-stack of 6 hepatocyte nuclei and 6 plasma cell nuclei were acquired for deconvolution processing and 3D modelling. As this was a tissue section, many of the cells were sliced open and so contained only partial nuclei. However, the 12 cells used in this study were specifically chosen because they had their nuclei wholly within the section thickness (as determined by visual inspection of the *Z*-series of 2D images looking to see if both the upper and lower vertices of the nucleus come into focus at some point in the series) and so could be completely reconstructed. Deconvolution of the *Z*-stacks was performed using the bright field Lucy-Richardson method via the freely available BiaQIm image processing software suite [9]. The point spread function of the microscope was measured using the novel method previously published [8].

### 2.3. 3D Modelling and Measurements

The deconvolved datasets represented 3D information of the whole tissue section thickness in the region of the selected cells (nucleus, cytoplasm, and extracellular matrix). They were then processed further to produce the 3D models of nuclear chromatin studied in this paper. The aims of this post-processing were to isolate the nucleus from extranuclear structures, uniformly enhance the contrast of the chromatin across the volume of the nucleus to facilitate qualitative and quantitative analysis and standardise the shapes of the nuclei (without affecting their overall volume) so that the comparative chromatin texture analyses will have fewer sources of potentially artefactual variation (i.e., to compensate for irregular nuclear shrinkage due to fixation and processing). In detail, these postprocesses are as follows.

1. Nuclear isolation: the nuclear structure was isolated by manually tracing around the nuclear profiles in 3D and discarding all extranuclear structural detail.
2. Shape standardisation: map the nuclear volume to a standard sphere of equal volume to the original nucleus.
3. Contrast standardisation: contrast was made more uniform across the volume by standardising the mean and SD of the greyscale distributions across all 2D slices that make up the 3D volume of the nucleus.
4. Contrast enhancement for visualisation: the contrast was then enhanced by 3D histogram equalisation followed by square root transformation of intensities.

Once postprocessed as above, the 3D nuclear chromatin datasets were subjected to quantitative chromatin texture analysis using the 3rd moment of Hu texture metric [10]. In order to compare the difference between 2D and 3D texture analysis on the same cells, this same texture metric was also calculated on a single central 2D slice through each of the nuclei.

The postprocessed nuclear volumes were also converted into fully interactive 3D models for qualitative inspection in the virtual reality modelling language (VRML) using the freely available FATCAM software package [11]. The models generated by FATCAM allow the user to fly into the heart of the nucleus and examine the internal structure from within as well as by the usual external rotational views. The aims of the qualitative inspections were to look for notable differences in the 3D internal structure and connectivity of the chromatin strands between plasma cell and hepatocyte nuclei and to elucidate the 3D structural means by which the chromatin distribution in plasma cell nuclei so often resembles a “clock face” when projected onto a 2D plane (as in cytology preparations) or cut into partial volume slices (as seen in histology preparations) despite this projection and slicing occurring seemingly at any random orientation.

## 3. Results and Discussion

**Figure 1:**
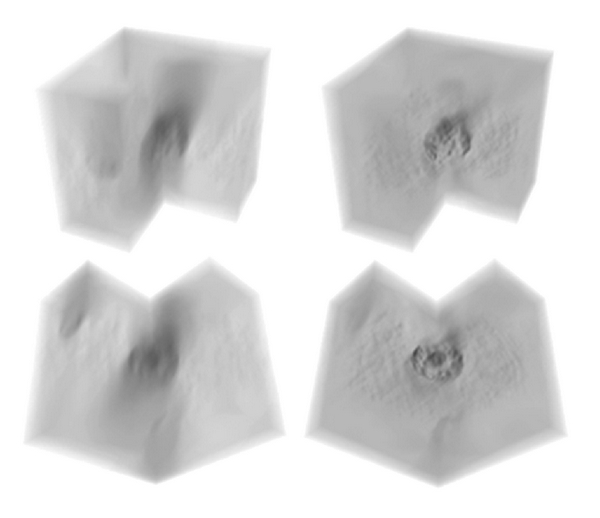
Partial volume of tissue including a whole hepatocyte nucleus before (left) and after (right) deconvolution. The volume in both cases is shown from two different angles (top and bottom). In the predeconvolved volume the smearing of structural details in the *Z*-direction prevents detailed structural analysis. The brightfield deconvolution method developed for this work greatly reduces this smearing. However, the deconvolved cytoplasmic details partly obscure the view of the nuclear chromatin within. As the aim of the present paper is to make studies of the nuclear chromatin, the extraneous information of the cytoplasmic structure is manually dissected away from the digital images as part of the image postprocessing steps described in the text.

Figure 1 shows a 3D rendering of an *Z*-stack of a hepatocyte nucleus with surrounding cytoplasm and it has a quarter of the volume “cut-away” for clarity and is shown at two angles. On the left is the original *Z*-stack prior to deconvolution and on the right is the deconvolved volume showing the greater nuclear chromatin detail in 3D following deconvolution.

**Figure 2:**
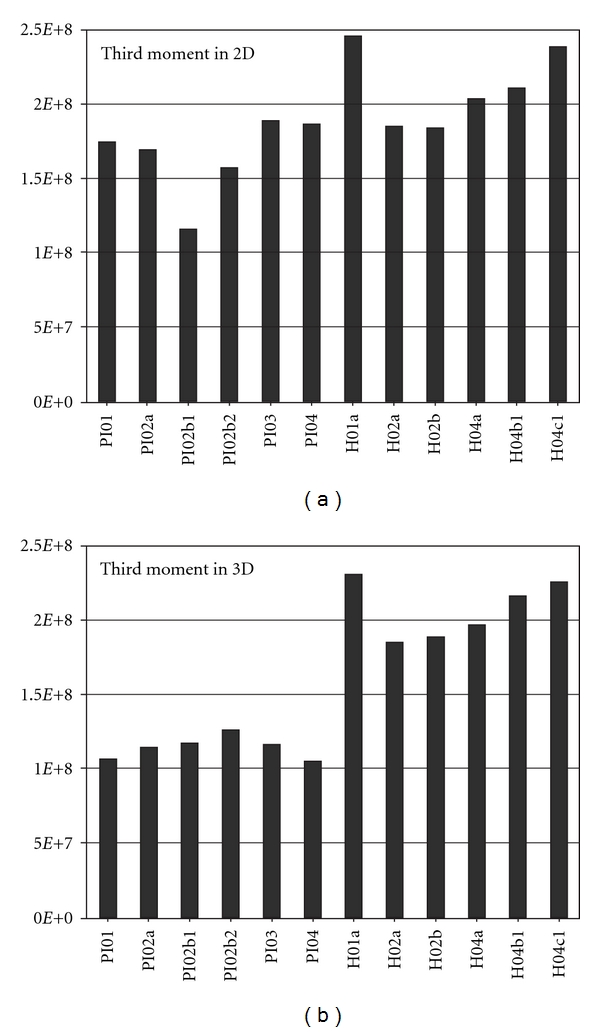
Chromatin texture measurement on all 12 cells used in this study by the third moment of Hu. The value of the third moment calculation is shown on the ordinate versus the cell measured on the abscissa (the 6 plasma cells followed by the 6 hepatocytes). The graph (a) shows the measurements as made on a 2D slice through the centre of each nucleus. The graph (b) shows the same calculation but performed on the whole 3D reconstructed nucleus. Clearly the measurement made on the 3D nucleus gives greater uniformity of the values between cells of similar type and greater distinction between different types of cell.

Figure 2 shows the results of the quantitative measurements of chromatin texture on all 12 cells. The difference between performing this measurement on the whole 3D volume as compared to a single central 2D section through each nucleus is that the 3D measurement makes the distinction between the two types of nuclei more obvious (for 3D measurements the Mann-Whitney *P* value is <0.01, whereas it is <0.05 for the 2D measurements). This also makes intuitive sense as in 3D one measures the chromatin distribution across the whole nucleus and so, for measurements which are insensitive to orientation, this will be the same for a given nucleus. However, when making this measurement on a 2D section through a nucleus, the value will be insensitive to the orientation of that section but can be expected to be different for any other section taken through the same nucleus at a different level or angle. Thus making measurements on 2D sections or projections bring with them an inherent level of variability that may be viewed as “noise” superimposed on the “signal” which does not exist if the measurement is made on the whole 3D structure. This, in turn, makes discrimination between different morphological groups more difficult if one restricts measurements to the 2D case (as with routine tissue sections or cytology preparations). This also has similar implications for the qualitative visual discrimination of 3D patterns from 2D samples as elaborated in the next paragraph.

**Figure 3:**
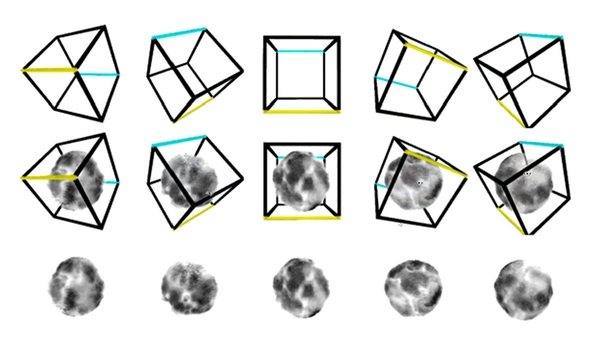
The freedom to view cells in 3D, as provided by the reconstruction methods in this paper, gives us greater morphological discriminatory abilities such that we can detect when cells that look different in 2D projections or slices (such as in cytology and histology preparations) are actually more similar than they first appear. This figure shows just one single plasma cell nucleus from different 3D angles. The angles are shown by the orientation of the boxes. In the top row, we see the box viewed from the different angles. In the bottom row we see the nucleus viewed from those same angles and in the middle row the two are superimposed. While the box viewed from different angles shows many different shapes we intuitively recognise this as being the same (or a very similar) object viewed from different angles. Because we have no such intuitive experience of complex nuclear chromatin, we cannot readily appreciate that the nuclei at the bottom are also identical and so they appear to us as different nuclei.

Figure 3 shows the results of one of the qualitative experiments where a single plasma cell nucleus, being reconstructed in 3D, is subsequently viewed in 2D projection from various angles. This is analogous to a cytology preparation of a whole cell lying on a slide—any given cell may be oriented on the slide in a random orientation. From this figure it is clear that, even though the cell is one and the same, it appears (from the cytologist's viewpoint) as if we are actually looking at 5 different cells because the 2D projection pattern of the same 3D chromatin arrangement is different depending on the angle of projection. For simple geometrical shapes (such as a cube) or familiar 3D forms (such as a cup or a face) we can intuitively recognise the 3D object from a multitude of its projections—the cubical frame at the top of Figure 3 will be obvious to most observers as the projection of the same 3D object from different angles even though the actual arrangement of the lines differs in the various projections. However, this intuitive sense is not present when one considers unfamiliar and irregular 3D objects such as the chromatin of the interphase nucleus. This has another implication in reverse. That is to say, if we had the freedom to view nuclear chromatin from all angles (such as is provided via the 3D imaging methods described in this paper) and we had 5 separate cells in a sample with identical chromatin (which might be neoplastic clones of each other, e.g.) then we could identify that those cells were identical (and hence possibly neoplastic). However, if we were restricted to seeing those cells fixed as they lie on a cytology slide at all different angles we would see 5 different 2D projection chromatin patterns similar to those shown in Figure 3 and we could not know they were identical. Thus the freedom to examine cells in 3D can enhance our morphological discriminatory abilities to detect both subtle differences between cells (Figure 2) and also similarities between cells (Figure 3) which could be of significant utility in comparative microanatomical studies.

As described in many classical texts, a very typical morphological feature of a mature plasma cell nucleus is its “clock face” or “cart wheel” chromatin pattern. This refers to the appearance of peripheral separate clumps of chromatin against the nuclear membrane (the “numerals” of the clock face) which often have a tapering extension facing radially inwards (the “spokes” of the cart wheel) with a central clump (the hub of the cart wheel or the centre of the clock face where the hands attach). Whereas these descriptors refer to a typical 2D pattern, the fact that this pattern is so characteristic implies a 3D structure that can display this pattern from almost any angle of projection (the orientation of plasma cells on a slide is not uniform yet they characteristically project this similar pattern) as well as being typical even when the plasma cell nucleus is cut in partial section through any plane at any angle (as is the case with histological preparations). This raises the interesting question of which 3D structure can give rise to a similar 2D pattern under such a diverse set of projections and partial sections through any angle? After studying the 6 plasma cell nuclei in 3D (including “flying into” the nucleus and observation of chromatin from the inside in interactive VRML—see supplementary movie 1 of the supplementary material available online at doi: 10.1155/2012/898707 for an example of this type of exploration) I have attempted to summarise the 3D architectural basis for the characteristic “clock face” pattern. I must emphasise that what follows is a stylised simplified representation of the chromatin but does capture the essence of the 3D arrangement of the plasma cell chromatin for the purpose of explaining this morphological feature.

**Figure 4:**
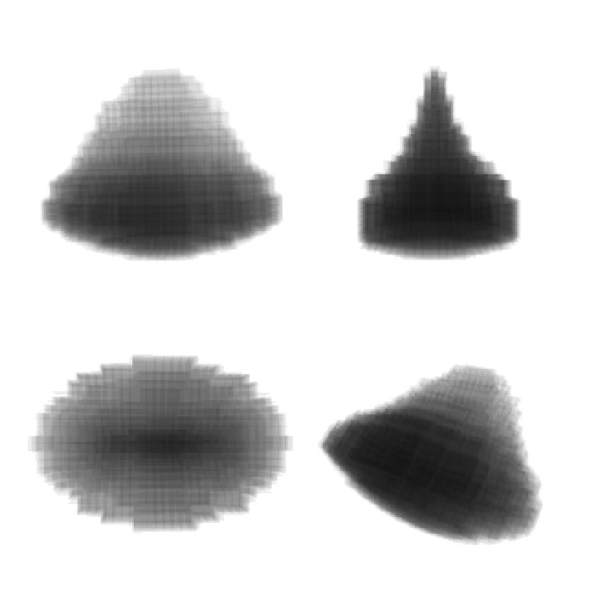
The basic chromatin structural unit of the plasma cell nucleus (the “clump unit” described in the text). This is essentially an ovoid curved cone. Side views (at 90 degrees to each other) are shown on the top and a plan view is shown bottom left. An oblique angle view is shown bottom right. This shape, in the arrangement shown in Figure 5, can explain the characteristic “clock-face” appearance to plasma cells in tissue sections and cytology preparations.

The chromatin in the mature plasma cell was found to be arranged in a variable number of peripheral clump units arranged in rings around the periphery. These peripheral clump units are the basic units of the pattern. Importantly, and perhaps counter-intuitively given the prominence of the central spot to the clock-face pattern, there is no tendency for a central clump (a clump at the 3D centre of the spheroidal nuclear space). In a detailed analysis, there are interesting connecting strands of chromatin in some plasma cell nuclei that pass through or near the centre (see supplementary movie 1) but these are not morphologically prominent as globular clumps neither are they a consistent feature in all plasma cells so do not take part in forming the characteristic central clump of the clock face pattern as seen in 2D projections/sections. The archetypal peripheral chromatin clump unit has an asymmetrical and curved conical shape shown in simplified form in Figure 4. This has a convex base applied to the nuclear membrane (the convexity follows the inner contour of the nuclear membrane) and a concave conical extension pointing towards the centre of the nucleus. The cross-section of this curved conical clump unit in a plane that is perpendicular to the base-apex axis of the cone is essentially elliptical such that at one angle of view the cone appears broad and blunt-tipped and at right angles to this plane it appears narrow and sharp tipped (Figure 4).

The arrangement of the clump units is as a series of intersecting peripheral rings with the clump units in each ring all arranged with their bases abutting the nuclear envelope and tapered ends pointing towards the centre (but with variable rotation about the base-tip axis of the conical clump units). The clumps in each ring are approximately regularly spaced with sparse chromatin in between them. The rings of clump units intersect at various angles to cover the periphery of the nucleus approximately evenly. Where they intersect, two rings share 2 clump units (one at each diameter of the intersection). This arrangement of the bases of the clump units on the nuclear membrane is analogous to the arrangement of the pentagonal black patches on the surface of a standard soccer ball. The typical “clock face” as seen in histology has between 4 and 8 clumps (on average about 5 to 7 clumps) but fewer clumps are seen in 3D (only about 4 or 5 clump units are present in a single circumference) which indicates that parts of clump units from above and below the central plane contribute to the clock-face “numerals” as seen in 2D projections.

**Figure 5:**
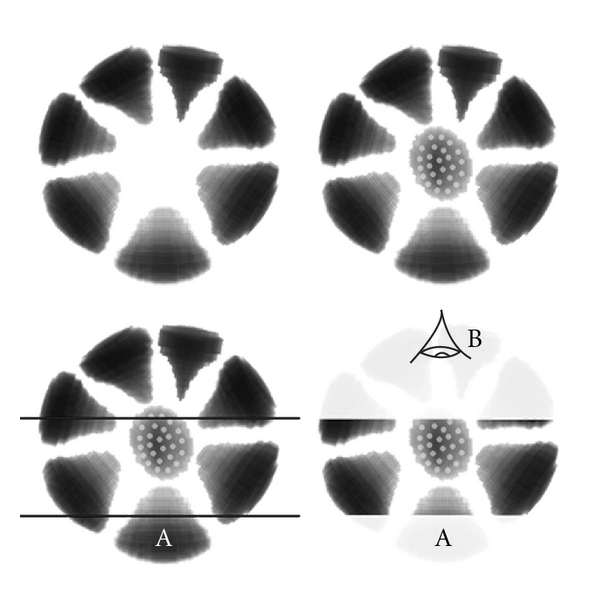
The plasma cell clock-face or cart-wheel nuclear pattern as seen in 2D sections/projections from almost any angle can be explained by the multiradial arrangement of peripherally placed clump units. Top left shows 7 such units in a circle (some of these 7 will actually derive from peripheral clumps slightly out of the plane of the diagram). There is no central clump but these rings of clump units are arranged at multiple angles around the nucleus in 3D so that, at any angle of projection, there is likely to be a clump unit directly centred above or below (or both) the viewing plane and this creates the appearance of a central chromatin blob when seen in 2D projections (top right: the stippled clump is present at the periphery of the nucleus but in a plane that is perpendicular to the plane of the diagram). The bottom two panels show that even if much of the nucleus is cut away (as in a thin histology section) and we view it from the plane of that section (viewer “B” looking “down” onto the section) then we will still see a “clock-face” arrangement of clumps. Here, part of the conical extension of the bottom-most clump in the figure (labelled “A”) will form the central clump to the clock face even though it is only partially present due to the conical nature of these clump units and their radial orientation. From the viewpoint of observer “B” the stippled clump is now one of the peripheral “numerals” in the clock face and there will be a similar clump radially opposed to it out of the plane of the figure (not illustrated).

As noted above, there is no tendency for a central clump in 3D space. The characteristic central “blob” in the clock-face/cart-wheel pattern seen in histology and cytology is actually a projected image of the peripheral clump unit directly above and/or below the viewing plane as shown in Figure 5. This 3D geometrical arrangement of clump units explains the various properties of the clock-face pattern as seen in 2D sections/projections.

1. Peripheral clumps are always present in a radial and equally spaced distribution no matter what angle of projection is seen and for most partial slice projections at any angle. This angle-of-projection invariance explains why the feature of a peripheral ring of clock face “numerals” is so characteristic regardless of projection angle or section thickness.
2. As the clump unit is conical there is nearly always some amount of central chromatin visible in the perpendicular axis of view even when the nucleus is not whole. This explains the characteristic feature of the central “hub” clump to the clock-face/cart-wheel.
3. As the conical clump units are ovoid in cross-section this explains why some clumps appear broad and blunt while others appear thin and tapered. Whilst this is not a feature of the “clock-face” motif it is a typical observation of plasma cells in tissue sections.

Supplementary movies 1 and 2 show fly-into sequences of the 3D structure of a typical plasma cell and hepatocyte nucleus. The grey levels have been inverted in these movies as this aids visual 3D depth cues but these are typical examples of the brightfield 3D data generated in this study. Note how the internal chromatin in the hepatocyte is characterised by more complex multiply branched internal connections in contrast to the plasma cell which has sparse and coarser internal connections. Also note the fine connections between peripheral clumps in the plasma cell which are not obvious from an external inspection as the great contrast of the large clumps throughout the nucleus superimposes on an external view. Thus the ability to fly “into” the nucleus and look at these clumps from the inside out reveals morphological detail not otherwise easily appreciable and is one of the advantages of the methods presented in this study.

Previous attempts at 3D chromatin structure evaluation have been described in the literature. Many of these use fluorescence microscopy and so are not applicable to routine brightfield staining so cannot be used to elucidate chromatin structure as we commonly see it in histology sections or cytology preparations and the structural resolution of the models has been crude relative to the methods used in the current work [12]. Brightfield 3D microscopy with routine stains has been achieved using a novel tomographic method [13]. However, this requires the individual cells to be isolated in a capillary tube for imaging and again the models are not as detailed as those presented here. Very detailed models have been generated using electron microscopy techniques [14] but these do not utilise routine brightfield stains and the altered tissue processing needed for EM all mean that the models obtained by those methods are not suitable for elucidating chromatin features as seen on routine histology preparations. In contrast to all of the above, the method presented in this paper can be used on routine histology preparations with ordinary brightfield light microscopy and has been demonstrated to give detailed 3D models of nuclear chromatin allowing microanatomical analysis of structures seen in everyday microscopy practice. While chromatin has been studied in this paper, the method may also be used to study cytoplasmic and extracellular detail. This type of analysis can explain the basis for morphological features seen in 2D tissue sections and can bridge the gap between light and electron microscopy in 3D reconstruction studies. Such enhanced 3D microanatomical knowledge can help explain the microanatomical basis for histophysiological and pathological processes and has been shown in this paper to enhance our morphological discriminatory abilities which are normally restricted by our 2D views of complex 3D structures at the microscopic level.

## 4. Conclusions

This paper has demonstrated the first results in human cells of a novel method of 3D brightfield light microscopy in the detailed imaging of nuclear chromatin with the formation of high resolution and fully interactive 3D models of the interphase nucleus. The method is applicable to routinely stained paraffin-embedded tissue sections. The models generated from 6 hepatocyte and 6 plasma cell nuclei were studied quantitatively and qualitatively and it was shown that the 3D information can give greater morphological discriminatory information than 2D sections/projections. Furthermore, study of the plasma cell chromatin in high resolution 3D resulted in a plausible microanatomical explanation of the characteristic clock-face nucleus that typifies plasma cells in 2D preparations.

## Supplementary Material

Supplementary Movie 1. This is a “fly-into” sequences of the 3D structure of a typical plasma cell nucleus. The grey-levels have been inverted to aid visual 3D depth cues but this is brightfield imagery. Note the prominent peripheral clumps of chromatin without a central clump. The internal chromatin connections are relatively sparse and coarse compared to those of a hepatocyte (see movie 2). Also note the fine connections between peripheral clumps which are not obvious from an external inspection as the dominant features of the large clumps throughout the nucleus superimpose on an external view. Thus the ability to fly “into” the nucleus and look at these clumps from the inside out reveals morphological detail not otherwise easily appreciable and is one of the advantages of the methods presented in this study.
Supplementary Movie 2. This is a “fly-into” sequences of the 3D structure of a typical hepatocyte nucleus. The grey-levels have been inverted to aid visual 3D depth cues but this is brightfield imagery. Note how the internal chromatin in the hepatocyte is characterised by more complex multiply-branched internal connections and is more dispersed in linear strands in contrast to the plasma cell.
